# Trends in Lifestyle-related Diseases and Their Risk Factors After the Fukushima Daiichi Nuclear Power Plant Accident: Results of the Comprehensive Health Check in the Fukushima Health Management Survey

**DOI:** 10.2188/jea.JE20210386

**Published:** 2022-12-05

**Authors:** Tetsuya Ohira, Hironori Nakano, Kanako Okazaki, Fumikazu Hayashi, Masanori Nagao, Akira Sakai, Mitsuaki Hosoya, Michio Shimabukuro, Atsushi Takahashi, Junichiro J. Kazama, Shigeatsu Hashimoto, Yukihiko Kawasaki, Hiroaki Satoh, Gen Kobashi, Seiji Yasumura, Hitoshi Ohto, Kenji Kamiya

**Affiliations:** 1 Radiation Medical Science Center for the Fukushima Health Management Survey, Fukushima Medical University, Fukushima, Japan; 2 Department of Epidemiology, Fukushima Medical University School of Medicine, Fukushima, Japan; 3 Department of Physical Therapy, Fukushima Medical University School of Health Sciences, Fukushima, Japan; 4 Department of Radiation Life Sciences, Fukushima Medical University School of Medicine, Fukushima, Japan; 5 Department of Pediatrics, Fukushima Medical University School of Medicine, Fukushima, Japan; 6 Department of Diabetes, Endocrinology and Metabolism, Fukushima Medical University School of Medicine, Fukushima, Japan; 7 Department of Gastroenterology, Fukushima Medical University School of Medicine, Fukushima, Japan; 8 Department of Nephrology and Hypertension, Fukushima Medical University School of Medicine, Fukushima, Japan; 9 Department of Endocrinology, Metabolism, Diabetology and Nephrology, Fukushima Medical University Aizu Medical Center, Fukushima, Japan; 10 Department of Diabetes and Metabolism, Juntendo University Urayasu Hospital, Chiba, Japan; 11 Department of Public Health, Dokkyo Medical University School of Medicine, Tochigi, Japan; 12 Department of Public Health, Fukushima Medical University School of Medicine, Fukushima, Japan; 13 Research Institute for Radiation Biology and Medicine, Hiroshima University, Hiroshima, Japan

**Keywords:** disaster, epidemiology, evacuation, lifestyle-related diseases, population studies

## Abstract

Residents were forced to evacuate owing to the radiation released after the Fukushima Nuclear Power Plant (NPP) accident following the Great East Japan Earthquake on 11/03/2021; thus, their lifestyles drastically changed. The Comprehensive Health Check (CHC) of the Fukushima Health Management Survey (FHMS) was performed to evaluate health statuses and prevent lifestyle-related diseases in evacuation area residents. The first part of the CHC survey is a retrospective analysis of pre- and post-disaster data on health check-ups of evacuation area residents. The second part is a cross-sectional, prospective analysis of post-disaster (fiscal year (FY) 2011–2017) data on health check-ups. Subjects were men and women living in 13 municipalities in areas surrounding the NPP in Fukushima Prefecture. Post-disaster (FY 2011–2012) overweight, hypertension, dyslipidemia, diabetes mellitus, metabolic syndrome, liver dysfunction, hyperuricemia, polycythemia and atrial fibrillation cases increased from the pre-disaster (FY 2008–2010) levels. This tendency was strongest among residents who were forced to evacuate. Proportion of overweight people remained unchanged, the prevalence of liver dysfunction decreased and the proportion of people with treated hypertension and dyslipidemia increased during FY 2011–2017. Meanwhile, the prevalence of diabetes mellitus and mean levels of HbA1c increased. Furthermore, Evacuees showed higher risks of diabetes mellitus, dyslipidemia, chronic kidney diseases and liver dysfunction than non-evacuees. Therefore, residents in the evacuation area, especially evacuees, are at high risk of developing lifestyle-related diseases, especially cardiovascular diseases; therefore, it is necessary to observe health statuses and implement measures to prevent lifestyle-related diseases.

## INTRODUCTION

On March 11, 2011, the Great East Japan Earthquake (GEJE) of magnitude 9.0 occurred off the Pacific coast of the Tohoku region of Japan. Owing to the massive tremors, tsunami, and fires throughout eastern Japan, 18,425 people were killed or missing in 12 prefectures, mainly in the Tohoku region. The tsunami that was generated by the earthquake hit the Fukushima Daiichi Nuclear Power Plant (NPP) located on the east coast of Fukushima Prefecture, causing an NPP accident. Due to the radiation released after the accident, many residents of Fukushima Prefecture who were living in areas surrounding the NPP were forced to evacuate. After the accident, the lives of more than 160,000 evacuees were deeply impacted.

In general, health problems are more likely to occur after disasters, whether natural or man-made. Many epidemiological studies have reported a higher incidence of psychiatric disorders, such as depression and post-traumatic stress disorder (PTSD), as well as cardiovascular diseases (CVDs), such as hypertension and myocardial infarction, after major disasters, such as earthquakes, tsunamis, hurricanes and terrorist attacks.^1^^–^^5^ Meanwhile, the duration of the health effects of disasters tend to vary depending on the type of disaster. For example, the GEJE caused significant damage in eastern Japan, especially in the Tohoku region, and the number of people who died directly during the GEJE was higher in Miyagi and Iwate Prefectures, which are located north of Fukushima Prefecture, than in Fukushima Prefecture, yet the number of disaster-related mortalities during the 10-year period after the disaster was reported to be more than twice as high in Fukushima Prefecture (2,319) than in Miyagi (929) and Iwate (470) Prefectures.^6^ This difference may be related to the NPP accident, resulting in a greater number of long-term evacuees in Fukushima Prefecture than in other prefectures. The number of evacuees who remained evacuated more than 10 years after the disaster were 28,147 from Fukushima Prefecture, compared to 3,555 and 822 from Miyagi and Iwate Prefectures, respectively.^7^

The Fukushima Health Management Survey (FHMS) was launched for the purpose of estimating external radiation doses and assessing the health statuses of Fukushima residents, which is necessary for the prevention, early detection, and treatment of diseases caused by the release of radioactive materials in residents who evacuated after the Fukushima NPP accident. The FHMS is categorized as a basic survey to assess external exposure doses and includes four additional detailed surveys, one of which is the Comprehensive Health Check (CHC).^8^^,^^9^ As a result of the GEJE and the Fukushima NPP accident, many residents were forced to live as evacuees, and their lifestyles, including dietary habits and exercise habits, changed drastically. The CHC was conducted to assess and address the health status of residents in evacuation areas with the aim to prevent lifestyle-related diseases and detect and treat diseases in their early stages.

In this study, we reviewed the results of longitudinal trends of lifestyle-related diseases except for cancer and their risk factors among residents in the evacuation area based on the CHC of the FHMS and discussed what measures can be taken in the future to prevent lifestyle-related diseases among residents in the evacuation area. Although the CHC investigated all age groups,^10^^,^^11^ this review summarizes the results of the survey for participants aged 40 years or older and considers the results for lifestyle-related diseases in comparison with data on health check-ups before the earthquake. We believe that the results of this survey will be useful for the health management of evacuees after future disasters that might occur in Japan and other parts of the world.

### Summary of the methods and results of the CHC

The CHC can be categorized into two parts. The first part consists of a retrospective analysis of pre- and post-disaster data on health check-ups of residents of the 13 municipalities that included evacuation areas. The second part consists of a cross-sectional and prospective analysis of post-disaster data on health check-ups of residents of the same municipalities. However, the target subjects of the first part of the survey were slightly different from those of the second part in each municipality because the first part was conducted using existing data for people aged 40 and older.

## METHODS AND RESULTS

### Survey using data on health check-ups performed before (fiscal year: FY 2008–2010) and after (FY 2011–2012) the disaster

#### Study population and design

The subjects of this survey were Japanese men and women aged 40 years and older living in 13 municipalities in areas surrounding the Fukushima Daiichi NPP in Fukushima Prefecture. Specifically, they were residents of Tamura City, Minamisoma City, Kawamata Town, Hirono Town, Naraha Town, Tomioka Town, Kawauchi Village, Okuma Town, Futaba Town, Namie Town, Katsurao Village, Iitate Village and Date City. In 2010, the total population of these municipalities was 278,276.

**Figure 1.**
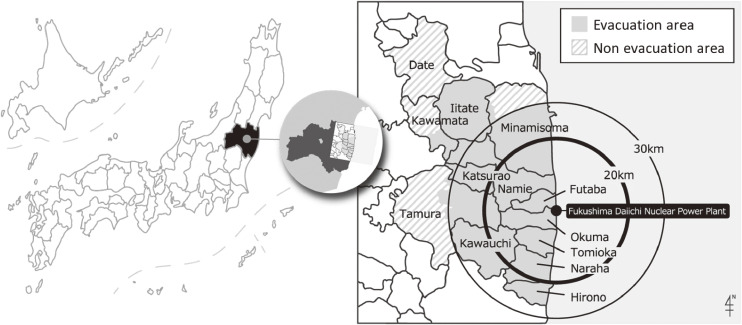
Geographic details of the evacuation area, non-evacuation area and Fukushima Daiichi Nuclear Power Plant.

After the disaster, the government designated these municipalities and parts of them as an evacuation area. As a result, all residents of Hirono Town, Naraha Town, Tomioka Town, Kawauchi Village, Okuma Town, Futaba Town, Namie Town, Katsurao Village and Iitate Village, as well as some communities in Tamura City, Minamisoma City, Kawamata Town and Date City, were forced to evacuate under the government’s direction (Figure 1, evacuation area). In this survey, we defined the areas in the municipalities that were not designated as evacuation areas as non-evacuation areas, and we compared data between the residents (non-evacuees) in those areas and the residents in the evacuation areas (evacuees). (Figure 1, non-evacuation area). In Date City, there were several spots with high radiation doses, and only a small number of people living in these limited areas were designated as residents who should be evacuated. However, since most of the residents in Date City were not evacuated and it was difficult to extract the designated evacuation areas, the entire city was treated as a non-evacuation area for the analysis.

In Japan, a system of Specific Health Check-ups and Specific Health Guidance focusing on the assessment of metabolic syndrome (MetS) was launched in 2008 for residents between the ages of 40 and 74 years. As part of this system, each municipality conducts annual health check-ups for those insured by the National Health Insurance (NHI; farmers, fishermen, and private business owners, among others) and for the elderly aged 75 years and over. Therefore, municipalities that are the target of this survey have previously conducted similar medical check-ups since 2008 (91,554 men and women were eligible for medical check-ups in 2010). In this survey, all analyses were limited to data on men and women aged 40–90 years. In 2010, the census population of people aged 40–90 years in these areas was 164,714. Among them, a total of 41,633 eligible residents (18,745 men and 22,888 women, average age 67 years) participated in health check-ups. The participation rate for the census population, including those who were not insured by the NHI, was 25.3%, and the participation rate for eligible individuals for check-ups (ie, those insured by NHI) was 45.5%.^12^

The post-disaster survey was conducted from June 2011 using a combination of the FHMS CHC survey and health check-ups conducted by the municipalities; the methods of the CHC and FHMS have been reported elsewhere^8^^,^^9^ and are briefly described below. According to the CHC of the FHMS, annual health check-ups have been conducted among residents of all ages in these municipalities since 2011 (and continue today). Individuals <15 years old received health check-ups at designated medical institutions in or outside of Fukushima Prefecture. Individuals aged ≥16 years who resided in Fukushima Prefecture received (1) health check-ups with additional items in Specified Health Check-ups conducted by each municipality or (2) group health check-ups conducted by Fukushima Medical University or (3) individual health check-ups at designated medical institutions in Fukushima Prefecture. Out-of-prefecture residents received (1) health check-ups with additional items in Specified Health Check-ups conducted by each municipality or (2) individual health check-ups at designated medical institutions outside of Fukushima Prefecture. The post-disaster survey was conducted nationwide because subjects had been evacuated to different parts of Japan. For the comparison before and after the disaster, we used (1) and (2) of the above CHC survey for residents aged 40 years and older in Fukushima Prefecture and (1) in the survey outside of the prefecture, which were measured by mostly the same laboratories as the health check-ups before the disaster. As a result, in the survey on trends among the overweight, 27,486 people (12,432 men and 15,054 women, 66% follow-up rate) received health check-ups after the disaster, and the average follow-up period was 1.6 years.^12^ The same method was used for surveys on polycythemia,^13^ hypertension,^14^ dyslipidaemia,^15^ diabetes,^16^ liver dysfunction,^17^ MetS,^18^ and other disorders, but there were some differences in the number of subjects. Informed consent was obtained from community representatives to conduct an epidemiologic study based on the guidelines of the Council for International Organizations of Medical Science. This study was approved by the Ethics Committee of Fukushima Medical University (#1916).

#### Measures and definitions

Height in stocking feet and weight in light clothing were measured, and body mass index (BMI) was calculated as weight (kg)/height (m^2^). In persons aged 40–75 years, waist circumference was measured. Abdominal obesity was defined as a waist circumference of 85 cm or more for men and 90 cm or more for women, which is the standard definition for Japanese. Overweight was defined as a BMI ≥25 kg/m^2^. Systolic blood pressure (SBP) and diastolic blood pressure (DBP) were measured on the right arm of seated participants. Hypertension was defined as SBP ≥140 mm Hg, DBP ≥90 mm Hg, or the use of antihypertensive medication. A 12-lead electrocardiogram was obtained in the supine position, and atrial fibrillation was diagnosed when there was no P-wave activity and the ventricular response was irregular.

Venous blood was collected from subjects using a serum-separating tube to measure aspartate aminotransferase (AST), alanine aminotransferase (ALT), γ-glutamyl transpeptidase (γ-GT), triglycerides (TG), high-density lipoprotein (HDL) cholesterol, low-density lipoprotein (LDL) cholesterol, and hemoglobinA1c (HbA1c). Creatinine, estimated glomerular filtration rate (eGFR), and uric acid (UA) levels were measured in some limited municipalities before the disaster and in all municipalities after the disaster. Additional measurements included fasting plasma glucose levels and the presence of protein, sugar, and occult blood in the urine. Liver dysfunction was defined as AST ≥40 IU/L, ALT level ≥40 IU/L, or γ-GT ≥50 IU/L. Diabetes mellitus was defined as an HbA1c (NGSP) level ≥6.5%, a fasting plasma glucose level ≥126 mg/dL, or the use of an antihyperglycemic medication. Hypo-HDL and hyper-LDL cholesterolemia were defined as HDL cholesterol <40 mg/dL and LDL cholesterol ≥140 mg/dL, respectively. Moreover, hyper-triglyceridemia was defined as fasting serum TG ≥150 mg/dL or being treated for dyslipidemia. Diagnosis of MetS was based on the definition of the Japanese Committee for Establishing Diagnostic Criteria for MetS, which is as follows: visceral obesity (waist circumference ≥85 cm in men and ≥90 cm in women) and the presence of at least two of three abnormalities (fasting serum TG ≥150 mg/dL and/or HDL cholesterol <40 mg/dL or being treated for dyslipidemia; SBP ≥130 mm Hg and/or DBP ≥85 mm Hg or being treated for hypertension; and fasting plasma glucose ≥110 mg/dL or being treated for diabetes). Hyperuricemia and was defined as a serum UA level of ≥7.0 mg/dL.

Venous blood was collected from subjects using ethylenediaminetetraacetic acid dipotassium as an anticoagulant for measuring erythrocyte counts in pre- and post-disaster surveys and leukocyte counts and their fractions in the post-disaster survey. Red blood cell count, hemoglobin level, hematocrit level and number of total white blood cells (WBCs), lymphocytes, monocytes, neutrophils, eosinophils, and basophils were determined using an automated cell counter. The definition of polycythemia was Hb ≥18.0 g/dL in men and ≥16.0 g/dL in women.^13^

Data on the history of cigarette smoking and weekly alcohol intake of participants ≥20 years old was obtained by a trained interviewer. Participants who reported consuming ≥44 g ethanol per day at the time of the survey were classified as current excessive drinkers. An interviewer also obtained information on lifestyle factors (eg, physical activity, dietary behaviors, and sleeping conditions) as well as medical history. Physical activity included regular exercise and daily walking or equivalent physical activities. Dietary behaviors included eating speed, frequency of skipping breakfast, eating dinner late, eating between meals, or eating bedtime snacks. Sleep conditions were evaluated based on the participants’ subjective perception of sufficient sleep.

#### Statistical analysis

In this study, subjects were divided into two groups, ‘evacuees’ and ‘non-evacuees’, and changes in lifestyle-related diseases were compared before (FY 2008–2010) and after (FY 2011–2012) the disaster. After linking the pre- and post-disaster data with personal identification numbers, the paired *t*-test was used to compare the means of continuous variables over time, and the McNemar test was used to compare proportions of categorical variables over time. A comparison between evacuees and non-evacuees regarding changes in continuous variables over time and differences in proportions of categorical variables over time were calculated using analysis of covariance or logistic regression models adjusted for age (years) and sex. For participants who were free of each disease at the baseline survey, the odds ratios (ORs) or hazard ratios (HRs) and 95% confidence intervals (CIs) of having each disease during follow-up were calculated using logistic regression models or Cox proportional hazards models after adjusting for age and other potential confounders. Data were analyzed with SAS statistical software package version 9.3 or 9.4 (SAS Institute, Cary, NC, USA). All probability values for statistical tests were two-tailed, and a *P* value of 0.05 or greater was considered statistically significant.

#### Trends in lifestyle-related variables among evacuees and non-evacuees before (FY 2008–2010) and after (FY 2011–2012) the disaster

Following the disaster, the prevalence of overweight/obesity,^12^ hypertension,^14^ hypo-HDL cholesterolemia,^15^ diabetes mellitus,^16^ liver dysfunction,^17^ MetS,^18^ atrial fibrillation,^19^ and polycythemia^13^ increased significantly in the 13 municipalities, especially among evacuees. Among evacuees, the proportion of overweight people increased from 31.5% to 38.8%, and the prevalence of hypertension, dyslipidemia, diabetes mellitus, and polycythemia also increased from 54.0%, 42.5%, 9.3%, and 0.89% to 60.1%, 55.4%, 12.2%, and 1.54% before and after the disaster, respectively. The prevalence of atrial fibrillation increased significantly from 1.9% to 2.4% before and after the disaster, respectively, regardless of whether the participants were evacuated.

Table 1 shows trends in the mean levels or proportions of lifestyle-related variables among evacuees and non-evacuees before (FY 2008–2010) and after (FY 2011–2012) the disaster. Changes in the mean levels of BMI, blood pressures, waist circumference, triglycerides, HDL cholesterol, fasting plasma glucose, and hemoglobin before and after the disaster were greater in evacuees compared with non-evacuees. Changes in the proportion of overweight people and prevalence of liver dysfunction before and after the disaster were also greater in evacuees compared with non-evacuees.

**Table 1.** Trends in mean levels or proportions of lifestyle-related variables among evacuees and non-evacuees before (FY 2008–2010) and after (FY 2011–2012) the disaster in the evacuation area of Fukushima Prefecture

	Non-evacuees				Evacuees					
	
	Before	After	Δ	*P* value	Before	After	Δ	*P* value	*P* value^d^	Reference
Men										
*n*	8,157				4,275					
Body mass index (BMI), kg/m^2^	23.5	23.7	0.2	<0.001	23.8	24.5	0.7	<0.001	<0.001	14 ^a^
Overweight (BMI ≥25 kg/m^2^), %	29.0	31.9	2.9	<0.001	32.8	42.6	9.8	<0.001	<0.001	14 ^a^
*n*	2,977				1,538					
Systolic BP, mm Hg	121.5	126.1	4.6	<0.001	121.5	127.3	5.8	<0.001	<0.01	15 ^b^
Diastolic BP, mm Hg	74.6	76.7	2.1	<0.001	74.4	77.8	3.4	<0.001	<0.001	15 ^b^
*n*	2,443				1,254					
Waist circumference, cm	82.5	82.8	0.3	—	83.1	85.1	2.0	—	<0.001	19 ^b^
Triglycerides, mg/dL	103.7	112.8	9.1	—	101.9	120.0	18.1	—	<0.001	19 ^b^
HDL cholesterol, mg/dL	58.4	58.4	0.0	—	59.1	56.3	−2.8	—	<0.001	19 ^b^
Fasting glucose, mg/dL	96.0	96.0	0.0	—	94.0	97.0	3.0	—	<0.001	19 ^b^
*n*	1,002				969					
Uric acid, mg/dL	5.5	5.6	0.1	—	5.4	5.6	0.2	—	—	21 ^a^
Creatinine, mg/dL	0.8	0.8	0.0	—	0.8	0.8	0.0	—	—	21 ^a^
*n*	1,349				3,278					
Hemoglobin, g/dL	14.46	14.57	0.11	<0.001	14.77	15.05	0.28	<0.001	<0.001	13 ^a^

Women										
*n*	9,658				5,396					
Body mass index (BMI), kg/m^2^	23.3	23.5	0.2	<0.001	23.5	24.0	0.5	<0.001	<0.001	14 ^a^
Overweight (BMI ≥25 kg/m^2^), %	27.4	29.2	1.8	<0.001	30.5	35.9	5.4	<0.001	<0.001	14 ^a^
*n*	4,229				2,293					
Systolic BP, mm Hg	119.8	123.9	4.1	<0.001	119.6	124.0	4.4	<0.001	0.33	15 ^b^
Diastolic BP, mm Hg	72.5	74.2	1.7	<0.001	71.7	74.5	2.8	<0.001	<0.001	15 ^b^
*n*	2,977				1,538					
Waist circumference, cm	82.0	81.8	−0.2	—	82.3	82.7	0.4	—	<0.001	19 ^b^
Triglycerides, mg/dL	94.5	98.9	4.4	—	91.8	104.2	12.4	—	<0.001	19 ^b^
HDL cholesterol, mg/dL	63.2	62.9	−0.3	—	63.7	62.5	−1.2	—	<0.001	19 ^b^
Fasting glucose, mg/dL	94.0	94.0	0.0	—	91.0	94.0	3.0	—	<0.001	19 ^b^
*n*	1,489				1,329					
Uric acid, mg/dL	4.6	4.6	0.0	—	4.5	4.5	0.0	—	—	21 ^a^
Creatinine, mg/dL	0.7	0.7	0.0	—	0.7	0.7	0.0	—	—	21 ^a^
*n*	1,923				4,168					
Hemoglobin, g/dL	13.04	13.15	0.11	<0.001	13.18	13.40	0.22	<0.001	<0.001	13 ^a^

All^c^										
*n*	17,322				8,684					
Liver dysfunction (non-drinkers), %	8.7	10.4	1.7	<0.001	9.8	14.2	4.4	<0.001	<0.001	18 ^a^
Liver dysfunction (drinking ≤22 g of ethanol/day), %	13.1	13.9	0.8	0.03	12.5	18.9	6.4	<0.001	<0.001	18 ^a^
Liver dysfunction (drinking >22 g of ethanol/day), %	39.7	42.5	2.8	<0.001	44.0	48.8	4.8	<0.001	<0.001	18 ^a^

BP, blood pressure; FY, fiscal year.

^a^Target age: 40–90 years old.

^b^Target age: 40–74 years old.

^c^No analysis by sex.

^d^*P* value for comparing differences between the evacuee group and non-evacuee group regarding changes before and after the disaster.

#### Risk factors for lifestyle-related diseases before (FY 2008–2010) and after (FY 2011–2012) the disaster

**Figure 2.**
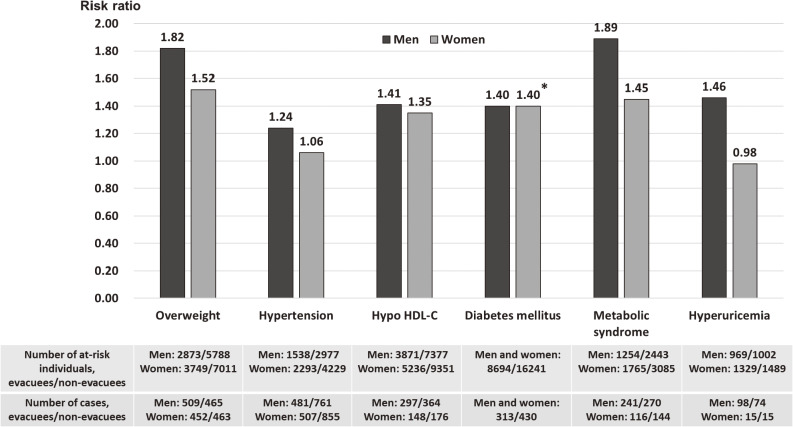
Multivariable-adjusted hazard ratios (HRs) or odds ratios (ORs) of overweight, hypertension, hypo-HDL cholesterolemia, diabetes mellitus, metabolic syndrome and hyperuricemia cases among evacuees compared with non-evacuees before (FY 2008–FY 2010) and after (FY 2011–FY 2012) the disaster. Overweight: body mass index ≥25 kg/m^2^; Hypertension: systolic blood pressure (SBP) ≥140 mm Hg, diastolic blood pressure (DBP) ≥90 mm Hg, or use of antihypertensive medication; Hypo HDL-C; HDL-C <40 mg/dL; Diabetes mellitus: HbA1c level ≥6.5%, fasting plasma glucose level ≥126 mg/dL, or use of antihyperglycemic medication; Metabolic syndrome: visceral obesity (waist circumference ≥85 cm in men and ≥90 cm in women) and the presence of at least two of three abnormalities (fasting serum triglycerides ≥150 mg/dL and/or HDL-C <40 mg/dL or medication use for dyslipidemia; SBP ≥130 mm Hg and/or DBP ≥85 mm Hg or use of antihypertensive medication; and fasting plasma glucose ≥110 mg/dL or use of antihyperglycemic medication); Hyperuricemia: serum uric acid level ≥7.0 mg/dL. Adjusted variables: age, smoking status, drinking status (all), body mass index (overweight, hypertension, hypo HDL-C, diabetes), waist circumference (metabolic syndrome, hyperuricemia), sex (diabetes), SBP (hypertension, hypo HDL-C, diabetes), alanine aminotransferase (hypo HDL-C, diabetes), γ-glutamyl transpeptidase (hypo HDL-C, diabetes), diabetes (hypo HDL-C), HDL-C (diabetes), LDL-C (diabetes), body weight change (metabolic syndrome), physical activity (metabolic syndrome, hyperuricemia), and sleep quality (metabolic syndrome). ^*^HR was calculated by using combined data of men and women for diabetes. FY, fiscal year; HDL-C, high-density lipoprotein cholesterolemia; LDL-C, low-density lipoprotein cholesterolemia.

As shown in Figure 2, in prospective analyses, evacuation was associated with an increased risk of overweight/obesity,^12^ hypertension,^14^ hypo-HDL cholesterolemia,^15^ diabetes mellitus,^16^ MetS^18^ and hyperuricaemia,^20^ and the effects of evacuation also appeared to be somewhat stronger in men than in women. Compared with non-evacuees, the multivariable-adjusted HR or ORs of evacuees with regard to overweight/obesity, hypertension, hypo-HDL cholesterolaemia, MetS, and hyperuricemia for men and women were 1.82 (95% CI, 1.61–2.07) and 1.52 (95% CI, 1.34–1.74), 1.24 (95% CI, 1.10–1.39) and 1.06 (95% CI, 0.94–1.18), 1.41 (95% CI, 1.20–1.67) and 1.35 (95% CI, 1.08–1.69), 1.89 (95% CI, 1.55–2.31) and 1.45 (95% CI, 1.10–1.92), and 1.46 (95% CI, 1.06–2.02) and 0.98 (95% CI, 0.45–2.12), respectively. Evacuation was significantly associated with the incidence of diabetes mellitus after adjusting for age, sex, and potential confounding factors, and the multivariable-adjusted HR for evacuees compared with non-evacuees was 1.40 (95% CI, 1.20–1.63). Furthermore, the incidence of liver dysfunction was significantly higher in evacuees than in non-evacuees, regardless of whether they consumed alcohol or not. The multivariable-adjusted OR for evacuees was 1.38 (95% CI, 1.20–1.58) for non-drinkers, 1.43 (95% CI, 1.29–1.59) for light drinkers, and 1.24 (95% CI, 1.09–1.42) for moderate and heavy drinkers.^17^ Meanwhile, evacuation was significantly associated with higher hemoglobin levels, even after adjusting for confounding factors, such as age, sex, smoking status, excessive ethanol intake, BMI, and baseline hemoglobin levels (β = 0.16, *P* < 0.001).^13^

As for factors other than evacuation, overweight, weight gain, and high BMI were associated with the incidence of hypertension, hypo-HDL cholesterolemia, diabetes mellitus, liver dysfunction, MetS, and atrial fibrillation. In addition, excessive alcohol consumption was associated with the risk of hypertension and atrial fibrillation; current smoking was associated with the risk of hypo-HDL cholesterolemia; and smoking cessation was associated with the development of abdominal obesity.^21^

### Survey using health check-ups data after the disaster (FY 2011–FY 2017)

#### Study population and design

The subjects comprised almost 210,000 men and women, except for foreign nationals, who were registered as residents in the evacuation area in Fukushima Prefecture between March 11, 2011 and April 1, 2012. This area included the above-mentioned 13 municipalities.

The methods of the CHC in the FHMS since 2011 have been described above. During FY 2011, the participation rate for the initial census population was 30.9% for people ≥16 years old.^22^ Informed consent was obtained from community representatives to conduct an epidemiologic study based on the guidelines of the Council for International Organizations of Medical Science. This study was approved by the Ethics Committee of Fukushima Medical University (#1319).

#### Measures and definitions

The measurements and definitions in the CHC are as described above. In the CHC, creatinine, uric acid, and white blood cell fractions were measured in addition to the usual items of the Specified Health Check-up for subjects aged 16 and over.

#### Statistical analysis

Changes in lifestyle-related diseases were compared between FY 2011–2012 and FY 2016–2017. After linking FY 2011–2012 data and FY 2016–2017 data with identification numbers, and we conducted the same analyses before and after the earthquake. The details of the analysis method are as described above.

#### Trends in lifestyle-related variables between FY 2011–2012 and FY 2016–2017^22^

Among participants in the CHC, those aged 40–90 years old (53,752 subjects, mean age 63.2 years) who had at least one visit during FY 2011–2012 were included in the analysis. Of those who had two or more check-ups during this period, the results of check-ups conducted in the year closest to the date of the disaster were used as the baseline, and the check-up data were compared with data of check-ups conducted in FY 2016–2017. Of the 53,752 subjects, 27,536 (12,254 men and 15,282 women, 51.2% follow-up rate, mean follow-up period 5.5 years) underwent health check-ups in FY 2016–2017.^22^

As shown in Table 2, from FY 2011–2012 to FY 2016–2017, the proportion of overweight people did not change, but the proportion of underweight people increased significantly. The proportion of hypertensive, antihypertensive medication use, diabetes mellitus, antihyperglycemic medication use, dyslipidemia, and medication use for dyslipidemia increased significantly. On the other hand, the mean values of SBP, DBP, and LDL cholesterol decreased significantly, whereas the mean value of HDL cholesterol increased significantly. In addition, the prevalence of liver dysfunction was significantly reduced. However, the mean value of HbA1c increased significantly.

**Table 2.** Trends in mean levels or proportions of lifestyle-related variables between FY 2011–2012 and FY 2016–2017 in the evacuation area of Fukushima Prefecture^22^

	FY 2011–2012	FY 2016–2017	Δ	*P* value
*n* ^a^	27,536			
Men, number (%)	12,254 (44.5)			
Overweight (BMI ≥25 kg/m^2^), %	33.1	33.4	0.3	0.10
Underweight (BMI <18.5 kg/m^2^), %	3.9	4.8	0.9	<0.001
Hypertension, %	54.0	60.0	6.0	<0.001
Antihypertensive medication use, %	38.4	48.2	9.8	<0.001
Systolic blood pressure, mm Hg	131.8	130.0	−1.8	<0.001
Diastolic blood pressure, mm Hg	78.5	73.8	−4.7	<0.001
Diabetes mellitus, %	10.9	16.0	5.1	<0.001
Antihyperglycemic medication use %	6.7	10.9	4.2	<0.001
Hemoglobin A1c, %	5.50	5.78	0.28	<0.001
Dyslipidemia, %	56.0	57.3	1.3	<0.001
Medication use for dyslipidemia %	20.5	30.2	9.7	<0.001
HDL cholesterol, mg/dL	59.5	61.5	2.0	<0.001
LDL cholesterol, mg/dL	124.8	119.3	−5.5	<0.001
Liver dysfunction, %	29.6	27.1	−2.5	<0.001

BMI, body mass index; FY, fiscal year.

^a^Target age: 40–90 years old, no analysis by sex and evacuation status.

#### Risk factors for lifestyle-related diseases after the disaster (FY 2011–2017)

In a cross-sectional study of health check-up data from the CHC within 1 year after the disaster, there were no regional differences in the distribution of WBC counts, such as neutrophils and lymphocytes,^23^ which indicated that there was no significant effect of radiation. In addition, a prospective study from FY 2011 to FY 2017 in the CHC reported that there were no significant associations of individual external radiation doses with WBC counts and the results of health check-ups.^24^

**Figure 3.**
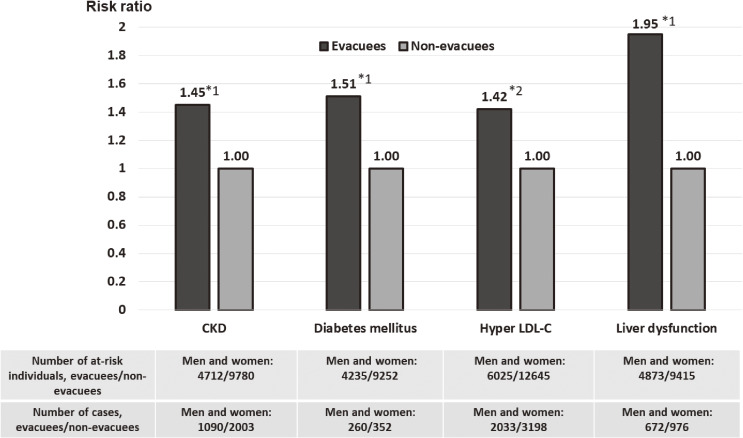
Multivariable-adjusted hazard ratios (HRs) or odds ratios (ORs) of CKD, diabetes mellitus, hyper-LDL cholesterolemia and liver dysfunction for evacuees compared with non-evacuees from FY 2011–2012 to FY 2013–2014 or FY 2013–2015. CKD: estimated glomerular filtration rate (eGFR) <60 mL/min/1.73 m^2^ or proteinuria; Diabetes mellitus: HbA1c level ≥6.5%, fasting plasma glucose level ≥126 mg/dL, or use of antihyperglycemic medication; Hyper LDL-C; LDL-C ≥140 mg/dL, or medication use for dyslipidemia; Liver dysfunction: AST ≥31 U/L, ALT ≥31 U/L, or γ-GTP ≥51 U/L. ^*^1 Multivariable-adjusted hazard ratio; ^*^2 Multivariable-adjusted odds ratio. Adjusted variables: age, sex, body mass index, smoking status (all), drinking status (diabetes, hyper LDL-C), hypertension (CKD), estimated glomerular filtration rate (CKD), diabetes (CKD, hyper LDL-C), dyslipidemia (CKD), body weight change (diabetes), physical activity (diabetes), and sleep quality (diabetes). CKD, chronic kidney disease; FY, fiscal year; LDL-C, low-density lipoprotein cholesterolemia.

Although a cross-sectional analysis revealed no association between evacuation and renal function within one year after the disaster,^25^ a 2.5-year prospective analysis, from FY 2011 to FY 2014, revealed that evacuation was a significant risk factor for incidence of chronic kidney diseases (CKDs; estimated glomerular filtration rate, <60 mL/min/1.73 m^2^ or proteinuria) even after adjusting for age, sex, baseline eGFR, obesity, hypertension, diabetes, dyslipidemia, and smoking, with an HR of 1.45 (95% CI, 1.35–1.56) for evacuees compared with non-evacuees (Figure 3).^26^ Similarly, a follow-up survey conducted for an average of 2.7 years, from FY 2011 to FY 2014, revealed that evacuees were at a higher risk of developing diabetes than non-evacuees: the multivariable-adjusted HR was 1.51 (95% CI, 1.28–1.79).^27^ Furthermore, the incidence of hyper-LDL cholesterolemia was 31% higher in evacuees than in non-evacuees between FY 2011–2012 and FY 2013–2015.^28^ The evacuation was significantly associated with the risk of hyper-LDL cholesterolemia after adjusting for age, sex, BMI, smoking habit, alcohol consumption, diabetes, weight change, sleep deprivation, and exercise, with an OR of 1.42 (95% CI, 1.32–1.52).^28^ The evacuation was also associated with an increased risk of liver dysfunction (AST ≥31 U/L, ALT ≥31 U/L, or γ-GTP ≥51 U/L). between FY 2011–2012 and FY 2013–2014, and the multivariable-adjusted HR was 1.95 (95%CI, 1.76–2.16).^29^ The prevalence of polycythemia was significantly higher among evacuees than non-evacuees, even 3–4 years after the disaster, regardless of the presence or absence of overweight/obesity, smoking, and hypertension.^30^ Meanwhile, regarding hypertension, the prevalence of hypertension peaked 1 year after the disaster, whereas the treatment and control of hypertension (SBP <140 mm Hg and DBP <90 mm Hg) increased thereafter.^31^

As mentioned above, the prevalence of liver dysfunction tended to improve 3–4 years after the disaster, and there was a significant association between improved liver dysfunction and improved daily physical activity and frequency of breakfast consumption.^29^ In addition, an active lifestyle was found to reduce the risk of developing dyslipidemia 3–4 years after the disaster.^32^ Furthermore, regarding associations between dietary patterns and the risk of lifestyle-related diseases, a Japanese vegetable diet pattern could reduce the risk of developing dyslipidemia and CKD.^33^^,^^34^

## DISCUSSION

In the CHC of the FHMS, we analyzed the health status of the residents in the 13 municipalities of Fukushima Prefecture based on the results of health check-ups and found that overweight, hypertension, dyslipidemia, diabetes mellitus, MetS, liver dysfunction, hyperuricemia, polycythemia, and atrial fibrillation increased in residents of the evacuation area after the disaster, compared with those before the disaster. This tendency was particularly strong among residents who were forced to evacuate. As a result of analyzing the survey data of these health conditions that worsened after the disaster up to 7 years after the disaster, the proportion of overweight people remained unchanged, and the prevalence of liver dysfunction decreased. As for hypertension and dyslipidemia, the mean levels of blood pressure and LDL cholesterol decreased and the level of HDL cholesterol increased as a result of an increase in the number of people taking medication, and this may contribute to the prevention of cardiovascular diseases. Meanwhile, the prevalence of diabetes mellitus and the mean levels of HbA1c increased. Furthermore, the increased risk of diabetes mellitus, dyslipidemia, CKD, and liver dysfunction was higher in evacuees than in non-evacuees. Therefore, residents in the evacuation area, especially evacuees, continue to be at a high risk of developing CVDs; therefore, it is necessary to continue observing their health statuses and implement measures to prevent CVDs.

### Comparison with surveys related to the GEJE other than the FHMS

A comparison of pre- and post-disaster health check-ups of tsunami evacuees from Soma City of Fukushima Prefecture revealed that the evacuees had worse BMI, abdominal circumference, HbA1c, and HDL cholesterol levels after the disaster.^35^ In a study of residents in Soma City and Minamisoma City, the proportion of those with diabetes and hyperlipidemia increased before and after the disaster, regardless of whether they had been evacuated or not.^36^ Furthermore, the increase in hyperlipidemia was greater in evacuees than in non-evacuees. The results of these surveys are not significantly different from those of the FHMS.^36^ Meanwhile, a study evaluating health check-up data among residents aged 40–74 years in Minamisoma City revealed an increasing trend for diabetes and a decreasing trend for hypertension from 2010 to 2017, regardless of the evacuation scenario, yet the risk of diseases was not significantly different between evacuees and non-evacuees in 2017.^37^ Since the health effects of the evacuation might be influenced by region-specific factors, it is necessary for the FHMS to consider regional factors in analyzing the long-term effects of evacuations in the future.

As mentioned above, the GEJE caused tremendous tsunami damage, mainly in the coastal areas of eastern Japan. In particular, many areas in Iwate and Miyagi Prefectures in the Tohoku region were severely damaged by the tsunami, and many residents were forced to leave their homes and move to temporary housing. To compare the incidence of diabetes mellitus after the disaster between those living in temporary housing and those living in other housing, a longitudinal follow-up study of 7,491 residents (mean age: 61.6 years) of coastal areas of Iwate Prefecture that were directly affected by the 2011 GEJE was conducted from 2011 to 2015. The results showed that, among men aged 64 years or younger, those living in temporary housing had a significantly higher risk of developing diabetes mellitus than those who did not live in temporary housing; however, there was no association between these factors among the women of the same resident population.^38^ Meanwhile, a longitudinal study of residents of the same target area but using data from 2011 to 2015, revealed that the risk of developing MetS was significantly higher among women aged 65 years or older who lived in temporary housing compared to those who did not live in temporary housing.^39^ Furthermore, weight gain after the disaster was more pronounced among temporary housing residents than among those who not residing in temporary housing.^40^ These results show a similar trend to the CHC results; therefore, prolonged evacuation appeared to have some effect on the development of lifestyle-related diseases.

### Interpretation and implication of the results of the CHC of the FHMS

Many epidemiological studies have reported an increase in the incidence of CVDs after natural disasters, such as earthquakes, tsunamis, and hurricanes.^1^^–^^4^ Potential contributing factors to this increase include worsening of medication status due to difficulties in receiving medical attention for chronic diseases, such as diabetes and hypertension, and the effects of acute psychological stress.^1^^,^^2^ Therefore, the effects of the disaster on CVD risk factors were generally observed within a few weeks to a few months. On the other hand, according to the CHC, an increase in lifestyle-related diseases, such as diabetes, continued more than a few years after the disaster, so long-term evacuation might have had an impact on the development of lifestyle-related diseases.

Although the mechanisms by which evacuation increases lifestyle-related diseases, especially CVD risk factors, are not fully understood, it is possible that weight gain observed in evacuees might have influenced an increase in these diseases. Weight gain reported in the post-disaster data from the CHC was significantly associated with an increased risk of hypertension, diabetes mellitus, dyslipidemia, liver dysfunction, CKD, polycythemia, and atrial fibrillation. Decreased physical activity in evacuees might also be associated with an increased risk of CVD. Many of the evacuees who were working before the disaster lost their jobs,^41^ were forced to leave their homes, and moved into cramped temporary housing, which might have reduced their opportunities for physical activity, such as farming and gardening. The resulting decrease in physical activity combined with the increase in body weight may have contributed to an increase in CVD risk factors. Indeed, according to the CHC, high physical activity was associated with improved liver dysfunction and a lower risk of developing dyslipidemia. Thus, health-related behaviors are an important factor in mediating the effect of evacuation on increased CVD risk.

In addition, the GEJE in Fukushima was a complex disaster that combined three events (major earthquake, tsunami, and NPP accident), which may have increased psychological burden among impacted populations. The proportion of ‘high psychological distress’ as assessed by the Kessler Psychological Distress Scale (K6) was higher in Fukushima residents than in Miyagi and Iwate residents, who were more affected by the tsunami but less affected by the NPP accident,^42^^–^^45^ and in Fukushima, evacuation, unemployment, and perception of radiation risks were associated with a high proportion of psychological distress. Furthermore, although the number of Fukushima residents with high psychological distress gradually decreased from 2011 to 2018, the percentage was still nearly twice as high as that in other regions in 2018. Therefore, prolonged evacuation after disaster may lead to longer-lasting psychological stress and an increase in stress-related diseases, such as MetS and diabetes.^46^ Further research is needed to confirm the correlation between lifestyle or psychological factors with the future incidence of CVDs, such as stroke and myocardial infarction, among Fukushima residents.

The reason for the increase in polycythemia among the evacuees may be the influence of lifestyle factors, such as obesity and smoking, as well as psychological stress. As a result of examining factors related to polycythemia among the evacuees from both physical and psychological aspects, we found no relationship between psychological factors, such as depression and trauma reactions, and polycythemia.^47^ Meanwhile, there was a significant association between the prevalence of polycythemia and lifestyle-related diseases and their risk factors, such as obesity, hypertension, diabetes, liver dysfunction, smoking, and excessive alcohol consumption.^47^ These results suggest that lifestyle-related diseases and their risk factors are involved in the increase of polycythemia among the evacuees.

### Strength and limitations

The strength of this study is that we analyzed data from all municipalities that were forced to evacuate after the Fukushima Daiichi NPP accident and included data from before and after the disaster. This allowed us to examine the effects of the disaster prospectively and to minimize the effects of regional bias. On the other hand, this study has some limitations. First, the participation rate of the CHC was not high and gradually declined over the years, so the results obtained in this survey may not be representative of all residents in the evacuation area. Second, the present study did not evaluate psycho-socioeconomic factors other than evacuation that may have affected the association between evacuation and lifestyle-related diseases. However, since the subjects of the CHC were the same subjects as those of the Mental Health and Lifestyle Survey of the FHMS, a combined analysis of the two is currently underway. For example, it was reported that PTSD-related symptoms were associated with MetS,^48^ job changes and unemployment were associated with liver dysfunction,^49^ and unemployment was associated with hyperuricemia.^50^ We believe that further prospective studies will clarify the association between evacuation and lifestyle-related diseases. Third, since we surveyed residents in the evacuation area only in the CHC, it is necessary to compare the results with areas that were not affected by the NPP accident and evacuation. Fourth, we have mainly studied the effects of evacuation but have not yet studied the effects of returning after evacuation. Finally, although this survey has more than 7 years of follow-up, the study period may be insufficient to examine long-term effects of evacuation on the incidence of cancer and CVDs, such as stroke and myocardial infarction. Therefore, further long-term follow-up surveys are needed to assess the long-term effects of evacuation and other factors.

### Conclusion

After the GEJE, the proportion of people with lifestyle-related factors such as obesity, hypertension, diabetes, dyslipidemia, liver dysfunction, MetS, polycythemia, or atrial fibrillation increased among residents of the evacuation area in Fukushima Prefecture, especially those who had been forced to evacuate. The effects of the evacuation were associated with increased CVD risk factors, such as diabetes and dyslipidemia, which persisted more than 7 years after the NPP disaster. On the other hand, after the earthquake, there was a decrease in the incidence rate of liver dysfunction, and mean levels of blood pressure and lipids decreased through improved treatment rates, and physical activity and a vegetable-based diet might reduce the incidence of lifestyle-related diseases. As of 2021, more than 35,000 Fukushima residents remain displaced. Thus, continuous prevention programs against lifestyle-related diseases should be implemented in cooperation with local governments and communities to prevent future lifestyle-related diseases, including CVDs and cancer, in post-disaster evacuees.
